# The Microbiota-Dependent Treatment of Wuzhuyu Decoction for Chronic Migraine Model Rat Associated with Anxiety-Depression Like Behavior

**DOI:** 10.1155/2023/2302653

**Published:** 2023-01-06

**Authors:** Nan Nan, Qi Wang, Mei-Jing Li, Yong-Song Xu, Xiao-Meng Guo, Rui Xu, Zhe Ma, Si-Hui Wang, Jing Li, Hui Zhao, Mu-Xin Gong

**Affiliations:** ^1^School of Traditional Chinese Medicine, Capital Medical University, Beijing 100069, China; ^2^Beijing Key Laboratory of Traditional Chinese Medicine Collateral Disease Theory Research, Beijing 100069, China; ^3^Shanxi University of Traditional Chinese Medicine, Shanxi 030619, China; ^4^Center for Endocrine Metabolism and Immune Diseases, Beijing Luhe Hospital, Capital Medical University, Beijing 101149, China; ^5^Beijing Key Laboratory of Diabetes Research and Care, Beijing 101149, China

## Abstract

We previously found that Wuzhuyu Decoction (WZYD) could affect central and peripheral 5-HT to relieve hyperalgesia in chronic migraine (CM) model rats, possibly related to gut microbiota. However, the exact role of gut microbiota has not been elucidated. Accumulating evidence points to the possibility of treating central nervous system disease via the gut-brain axis. In our study, the inflammatory soup-induced CM model rats presented depression- and anxiety-like behaviors which both related to insufficient 5-HT. It was found that antibiotic administration caused community dysbiosis, and proteobacteria became the main dominant bacteria. The bacteria related to short-chain fatty acids and 5-HT generation were reduced, resulting in reduced levels of 5-HT, tryptophan hydroxylase, and secondary bile acids. Functional prediction-revealed sphingolipid signaling pathway in CM rats was significantly decreased and elevated after WZYD treatment. The effect of WZYD could be weakened by antibiotics. The CM rats exhibited anxiety- and depression-like behavior with 5-HT and number of neurons decreased in the CA1 and CA2 regions of hippocampal. The treatment of WZYD could recover to varying degrees. Antibiotics combined with WZYD attenuate the effect of WZYD on increasing the 5-HT content and related protein expression in the brain stem, plasma and colon, reducing CGRP, c-Fos, and inflammatory factors. And antibiotics also led to colon length increasing and stool retention, so that the antimigraine effect was weakened compared with WZYD. This experiment revealed that gut microbiota mediated WZYD treatment of CM rats with anxiety-depression like behavior.

## 1. Introduction

A lot of studies have been made on the relationship between the gut microbiota and brain for decades; the bidirectional influence between the microbiota and nervous system disease and the modulation of gut microbiota by medicine has gained much attention in nervous system disease. Chronic migraine (CM) is a burdensome and disabling neurological disease, which presented one side or bilateral pulsating headache with nausea, vomiting, and other symptoms for more than 3 months. It is often associated with psychiatric comorbidities, such as depression [1]. Depressive symptoms were independently associated with migraine, and the risk of incident migraine was 4.13-fold higher than controls in the patients with depression [2], and incident major depression in migraine patients was more than 5-fold higher than in persons without migraine [3]. The risks of migraine chronification and spend in healthcare were increased in comorbidity depression [4].The depression- and anxiety-like behaviors were presented in inflammatory soup-induced chronic migraine rats [5]. Because of the effect of tricyclic antidepressants on migraine prevention, there may be a shared mechanism of serotonergic dysfunction between migraine and depression [6].

Wuzhuyu Decoction (WZYD), a traditional Chinese medicine compound, is used for the treatment and prevention of migraine. Previous research found that WZYD regulated central and periphery 5-hydroxytryptamine (serotonin, 5-HT) relief chronic migraine accompanied by changes in microbiota but without direct evidence of the relationship between them [7]. Moreover, WZYD has been shown to be effective in treating depression [8]. There are many researches and data on the relationship between gut microbiota and pathophysiology of neuropsychiatric and neurological disorders [9]. The gut microbiota influenced tryptophan (Trp) metabolism pathway and reduced 5-HT in both brain and gut, leading to the occurrence of migraine and depression [10].

Therefore, this study is aimed at observing the direct relationship between WZYD efficacy and gut microflora in chronic migraine associated with anxiety-depression like behavior, by using antibiotics to establish a germ-free migraine model. It might provide a new experimental basis for WZYD in treating migraine based on the gut-brain axis.

## 2. Materials and Methods

### 2.1. Animals and Drug

Fifty male Sprague–Dawley rats (200 ± 20 g) were kept in specific pathogen-free conditions with standard 22-24°C and 12 h light/dark cycle, which were applied from Beijing Vital River Laboratory Animal Technology Company. The rats could get sterilized food and water freely. This experiment procedures and ethical approval number were both approved by Beijing Capital Medical University Ethics Committee (No. AEEI-2016-021).

Inflammatory Soup (IS) was comprised of histamine (2 mmol/L; >97%), serotonin hydrochloride (2 mmol/L; ≥98%), prostaglandin (0.2 mmol/L; ≥93%), and bradykinin (2 mmol/L; ≥98%). The first three were purchased from Sigma-Aldrich (USA), and the last one was purchased from APExBIO (USA). Dissolve the above reagents in 10 mM 2-[4-(2-hydroxyethyl)piperazin-1-yl] ethanesulfonic acid (HEPES) (pH = 5.5; ≥99%, Solarbio Ltd., China).

The raw materials of WZYD were extracted, decompressed, concentrated, and dried into dry powder as previously reported [11]. There are four raw materials in WZYD, Euodiae Fructus, Ginseng Radix et Rhizoma, Jujubae Fructus, and Zingiberis Rhizoma Recens. The sources and amounts of raw materials and the contents of active ingredients were shown in Supplementary 1.

The antibiotic cocktail (ABX) was consisted of ampicillin (5 mg/mL, Macklin Inc., China), metronidazole (5 mg/mL, Macklin Inc., China), neomycin (5 mg/mL, Macklin Inc., China), and vancomycin (2.5 mg/mL, Macklin Inc., China) which dissolved in sterile water.

### 2.2. Surgical Procedure

After the rats were anesthetized, a single cannula (62001, RWD Co., Ltd., China) was placed into cranial window and attached to the skull and screws as previously mentioned [7]. Pay attention to maintain the dural integrity. The obturator cap (62101; RWD Co., Ltd., China) was installed onto the cannula to prevent blockage. After the surgery, all rats were returned to their individual cages until the sensory thresholds were recovered to the initiation of the experimental procedure.

### 2.3. Experimental Design

All rats were randomly divided into 5 groups with 10 rats in each group, including control, model, WZYD (1.686 g/kg·d), WZYD+ABX, and ABX (20 mL/kg·d) groups.

In briefly, the 10 *μ*L of HEPES buffer (pH = 7.4) or IS was infused onto dura through the cannula in the control group or the other groups. HEPES or IS stimulation was once every 3 days for 8 times from the cannula. The WZYD and WZYD+ABX group rats were administrated with WZYD (10 mL/kg) everyday. The WZYD+ABX and ABX group rats were given oral gavage of ABX every 12 h. The others were administered equal volume of vehicle. The rats were free in the whole process of experimental and back to their individual cages.

After 8 times of IS, the open field test and fresh fecal samples collection were operated. Then, the rats were sacrificed for plasma from the external jugular vein. All the rats were transcardially perfused with cold fresh saline. Five rats in each group were sacrificed, and specimens of trigeminal nucleus caudalis (TNC), rostral ventromedial medulla (RVM), periaqueductal gray (PAG), other brainstems, and hippocampus were quickly separated and collected for WB or ELISA. In addition, five other rats in each group were transcardially perfused with cold 4% paraformaldehyde after saline, then postfixed the specimens of brain and colon in 4% paraformaldehyde for immunostaining or Nissl staining.

### 2.4. Behavioral Test

The von Frey filament (Aesthesio, Italy) was used to determine the facial mechanism withdrawal threshold prior to the HEPES or IS injection. The filaments were pressed on the periorbital region buckled slightly until a positive response, such as draw back, yell, or scratching. The filament of positive response was recorded in three of five tests.

The heat plate (YLS-6BA, Shandong, China) was turned on and kept the temperature at 55°C. The rats were put on the heat plate instrument and ensured the feet contacting heat plate. The latency time was recorded until the rats licked or flicked their hind paw, jumped, and yelled. It was measured 3 times, and the average was taken as a thermal pain threshold. There was an interval of 5 min between each measurement.

Rats were examined for exploratory behavior and tension state by the open field test (OF, TrueScan 2.01, Softmaze, China) for depression and anxiety test. The rats were placed in the black box center (100 × 100 × 40 cm). In a quiet environment, rat activity paths were captured and timed. The total motor distance (total distance), total rest time (total rest time), the number of central region crossing (numbers of traveling inner), the percentage of the central region motor distance (inner distance: ID%), and the percentage of residence time in the central area (inner time: IT%) were recorded. After each animal experiment, 75% ethanol was used to wipe the box, in case of affecting the behavior of subsequent experimental animals.

### 2.5. ELISA Analysis

The procedure of ELISA analysis was conducted following the instructions of manufacturer. The colon, brainstem, and hippocampus were homogenized in ice-cold PBS. The supernatants were obtained after centrifugation. The contents of calcitonin gene-related peptide (CGRP) in plasma, 5-HT in plasma, brainstem, hippocampus and colon, and 5-hydroxyindoleacetic acid (5-HIAA) in brainstem were determined using the corresponding ELISA kits from Nanjing Jiancheng Int. (Nanjing, China).

### 2.6. Western Blotting

The PAG, RVM, TNC, and colon specimens were homogenized in PIRA lysis buffer (WB3100, NCM, China) containing 1% protease inhibitor cocktail (p001, NCM, China). Then, the homogenates were centrifuged at 12000 rpm for 20 min. The supernatant of dissolved proteins was collected and determined by BCA (bicinchoninic acid) kits (WB6501, NCM, China). The loading buffer (B1012, Applygen, China) was added to the unified proteins' supernatant in a 1 : 4 ratio. Proteins were separated in 8%, 10%, or 12% gradient gels via sodium and transferred to polyvinylidene fluoride membranes (PVDF). The nonspecific binding sites on PVDF were blocked with 5% nonfat milk (P1622, Applygen, China) for 2 h, and the membranes were washed 3 times and placed in primary antibodies overnight at 4°C. The molecular weight, No., brand, and dilution ratio of all primary antibodies were shown in Supplementary 2. Then, the membranes were washed 3 times and incubated with the horseradish peroxidase-conjugated secondary antibodies (SA00001-1 and SA00001-2, 1 : 10000, Proteintech, America; sc-2789, 1 : 10000, Santa Cruz, America) for 1 h at room temperature. The membranes were washed again for 3 times. The gel imaging system (Fusion, Germany) was used to detect antibody-reactive bands with chemiluminescence reagent kit (P10200, NCM, China), which were analyzed by ImageJ software.

### 2.7. Immunostaining

The postfixed PAG, TNC, and colon specimens were cut into sections of 10 *μ*m thick. The sections were incubated with primary and secondary antibodies, respectively. The DAB (3,3-diaminobenzidine) reagent or DAPI was used for immunohistochemistry (IHC) or immunofluorescence (IF) [12]. The pictures were captured by a panoramic scanner.

### 2.8. Nissl Staining

The 10 *μ*m thick sections of postfixed hippocampus specimens were stained in Nissl stain (G1036, Servicebio, China) for 3 min and rinsed [13]. They were differentiated with 0.1% ice vinegar until the Nissl body was dark blue with pale blue or colorless background. The slices were dehydrated in anhydrous ethanol and sealed with neutral resin. All the slices were observed with microscope (ECLIPSE Ti-U, Nikon, Japan), and Nissl-stained neurons were counted.

### 2.9. 16S rRNA Sequencing and Data Analysis

The fecal samples of the rats were got from -80°C ultralow temperature refrigerator. Firstly, microbial DNA of fecal samples was extracted using the DNA Kit (Omega Bio-tek, Norcross, GA, U.S.) as the manufacturer's protocols and determined purification by NanoDrop 2000 UV-vis spectrophotometer (Thermo Scientific, Wilmington, USA) and quality by 1% agarose gel electrophoresis. Secondly, The PCR system (GeneAmp 9700, ABI, USA) was used to amplify the V3-V4 hypervariable regions of bacterial 16S rRNA from feces with primers 338F (5′- ACTCCTACGGGAGGCAGCAG-3′) and 806R (5′-GGACTACHVGGGTWTCTAAT-3′). According to the following program: 3 min of denaturation at 95°C, 27 cycles of 30 s at 95°C, 30 s for annealing at 55°C, 45 s for elongation at 72°C, and a final extension at 72°C for 10 min, PCR reactions were consisted of 4 *μ*L of 5 × FastPfu Buffer, 2 *μ*L of 2.5 mM dNTPs, 0.8 *μ*L of each primer (5 *μ*M), 0.4 *μ*L of FastPfu Polymerase, and 10 ng of template DNA. The resulted PCR products were extracted and purified again. Finally, sequenced the PCR production on Illumina MiSeq platform (Illumina, San Diego, USA) according to the standard protocols.

Bioinformatic analysis was conducted to the sequenced results. The raw data of sequenced results were operated by QIIME2 system and obtained the feature table of amplicon sequence variant by quality filtered and trimmed, denoised, and merged, which was aligned with GREENGENES database. The operational taxonomic units (OTUs) were defined as the sequence identities above 97%. The analysis of alpha diversity was arranged for richness and diversity, such as Chao1 richness estimator, ACE index, Shannon diversity index, and Simpson diversity index. The analysis of beta diversity was visualized by principal component analysis (PCA) to reveal the microbiota variation among groups. According to the abundance, the microbial functions were predicted by phylogenetic investigation of communities by reconstructing unobserved states (PICRUSt), and pathways were analyzed by Kyoto Encyclopedia of Genes and Genomes (KEGG) and MetaCyc database. The methods include ANOVA and Kruskal-Wallis, and linear discriminant analysis effect size (LEfSe) was employed to identify the bacteria with different abundance among groups.

### 2.10. Statistical Analysis

Two-way analysis of variance (ANOVA) with Holm-Sidak's multiple comparisons test for the threshold or one-way ANOVA with Bonferroni's post hoc test for other experimental data was used after the normality and equal variance tests were passed. The Kruskal-Wallis test was used following Dunn's test, if the experimental data were nonnormality, or the variance was homogenous. All data analyses were using bilateral tests and *P* < 0.05 indicated statistical significance. The experimental data were shown as the mean ± standard error of mean (SEM). The GraphPad Prism 8 (GraphPad Software, San Diego, CA) was applied for statistical analyses and graph generation both [7].

## 3. Result

### 3.1. Role of Gut Microbiota in WZYD Improving Hyperalgesia and Anxiety-Depression Like Behavior

**Figure 1:**
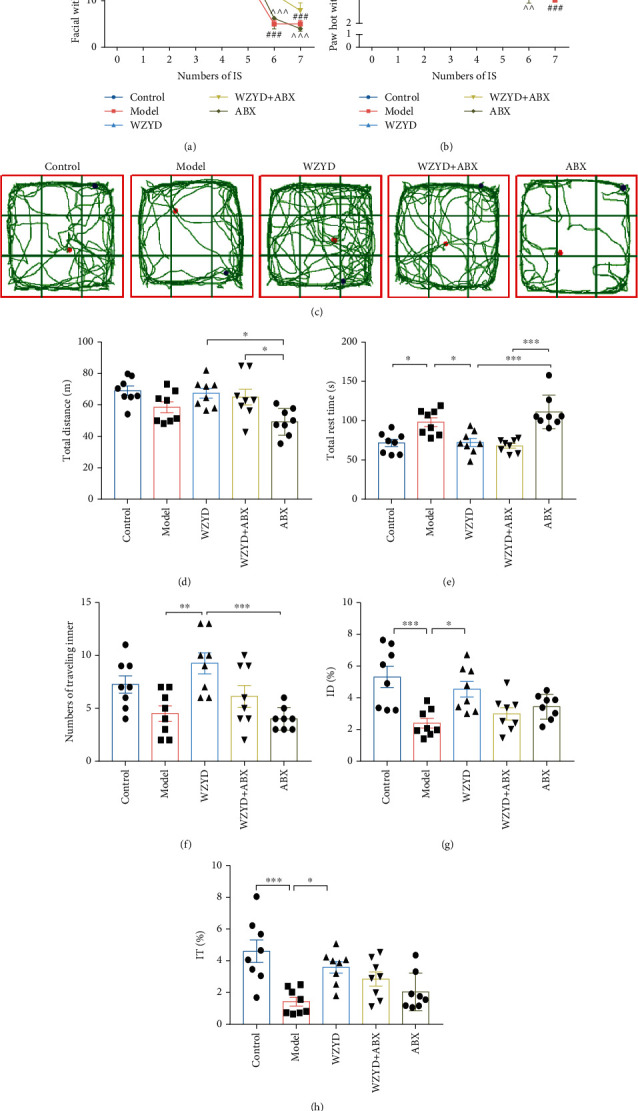
The gut microbiota mediated the effects of WZYD improving hyperalgesia and anxiety-depression like behavior. (a) Facial mechanism withdraw threshold. (b) Paw hot withdraw threshold. (c–h) The open field test. (c) The route of representative rat of five groups. (d) Total distance. (e) Total rest time. (f) Number of traveling inner. (g) Inner distance (ID%). (h) Inner time (IT%). ^∗^*P* < 0.05, ^∗∗^*P* < 0.01, and ^∗∗∗^*P* < 0.001.

The IS-induced chronic migraine model rats were used for WZYD treatment. The ABX acted to removed gut microbiota. The ABX combined with WZYD was used to observe the role of gut microbiota in WZYD preventing migraine chronification. The facial mechanical allodynia and plantar thermal hyperalgesia were significantly reduced after 5 times of IS administration in model group with a time dependent manner. Consistent with the previous study, WZYD showed the inhibitory effect of IS-induced reduction of pain threshold, which could be reversed by ABX (Figures 1(a) and 1(b)). There was no effect of ABX for chronic migraine.

After 8 times of IS administration, the model rats showed anxiety-depression like behavior (Figures 1(c)–1(h)). In the open field test (OF), total distance and total rest time were used to evaluate depression (Figures 1(d) and 1(e)), numbers of traveling inner, ID%, and IT% to anxiety (Figures 1(f)–1(h)). The total rest time of model group was significantly more than control group, and total distance travelled had a reduction tendency, which reflected depression. The treatment of WZYD improved depression-like behavior in CM rats and free-germ rats. The CM model rats demonstrated too afraid to explore and preferred in outer perimeter. The numbers of traveling inner, ID%, and IT% were reduced in model group and restored in WZYD group.

### 3.2. The Regulation of Gut Microbiota by WZYD and ABX Treatment

**Figure 2:**
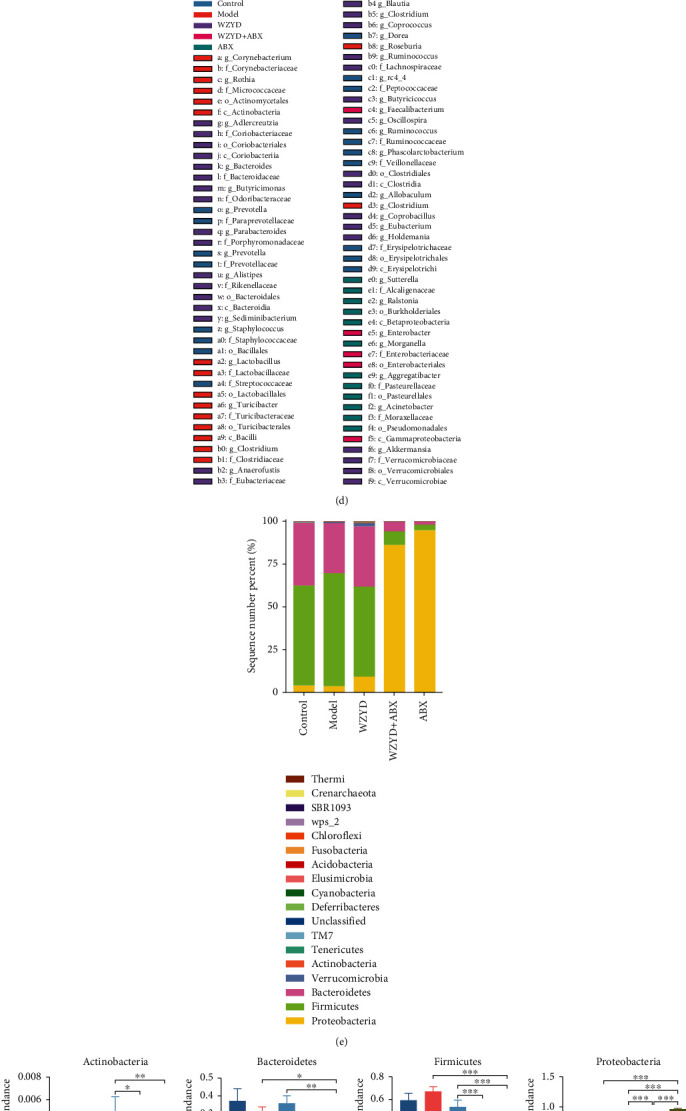
The effects of ABX intervention on WZYD regulating the gut microbiota in CM. (a) The rarefaction curve. (b) The alpha diversity analysis. (c) The beta diversity analysis with PCA. (d) The LEfSe analysis. (e) The abundance composition of phylum level. (f) Relative abundance of gut microbiota community members at the phylum level. (g) Relative abundance of gut microbiota community members at the genus level. ^∗^*P* < 0.05, ^∗∗^*P* < 0.01, and ^∗∗∗^*P* < 0.001.

The rarefaction curves of all groups were constructed and tended to be flat, which indicated that the sequencing depth was reasonable. It could be seen that the numbers of OTU were decreased in the WZYD+ABX and ABX groups (Figure 2(a)). The ABX intervention reduced the number of gut microbiota. The alpha diversity index in richness and diversity was analyzed in each group, which showed no significant differences among control, model, and WZYD groups. Compared with WZYD group, the Chao1 and ACE of richness index and Shannon and Simpson of diversity index were significantly lower after ABX treatment. The ABX intervention had an impact on the richness and diversity of microbiota, and WZYD administration tended to reverse richness without statistical difference (Figure 2(b)). There were some differences in PCA analysis at genus level. Although the control, model, and WZYD samples were not completely separated, the WZYD group was closer to the control group. The WZYD+ABX and ABX groups were significantly separated from the other groups at the first principal coordinates, indicating that ABX administration caused significant differences in the gut microbiota (Figure 2(c)).

The LEfSe analysis was performed for the gut microbiota at each level. The results showed that there were 1 class, 2 orders, 8 families, and 8 genera significantly different detected in control group; 2 phyla, 2 classes, 3 orders, 5 families, and 7 genera significantly different in model group; 2 phyla, 4 classes, 4 orders, 8 families, and 17 genera significantly different in WZYD group; 1 class, 1 order, 1 family, and 2 genera significantly different in WZYD+ABX group; and 1 phylum, 1 class, 3 orders, 3 families, and 5 genera significantly different in ABX group. The details were shown in Figure 2(d).

A total of Bacteroidetes, Firmicutes, and proteobacteria at the phylum level were exceeded at 97% (Figure 2(e)). Compared to the model group, the percentage of Firmicutes and Bacteroidetes were, respectively, 3.32% and 1.75%, which were significantly reduced (*P* < 0.05), while proteobacteria was increased significantly (*P* < 0.05, Figure 2(f)) in the ABX group.

The taxonomy of each group at genus level was analyzed and shown in Figure 2(g). Compared to the model group, the relative abundances of Acinetobacter (*P* < 0.01) and Butyricicoccus (*P* < 0.05) were significantly increased after WZYD treatment, the relative abundances of Enterobacter (*P* < 0.001), Sutterella (*P* < 0.01), and Morganella (*P* < 0.05) were significantly increased in the ABX group, and the relative abundances of Lactobacillus (*P* < 0.001), Prevotellaceae_Prevotella (*P* < 0.001), Rothia (*P* < 0.01), Blautia (*P* < 0.05), Roseburia (*P* < 0.01), Phascolarctobacterium (*P* <0.05), and Erysipelotrichaceae_Clostridium (*P* < 0.05) were decreased significantly in the ABX group. The above results indicated that WZYD treatment modulated the gut microbiota, and WZYD combined with ABX administration partially eliminated WZYD effect on the gut microbiota.

### 3.3. The Regulation of Gut Microbiota Function by WZYD and ABX Treatment

**Figure 3:**
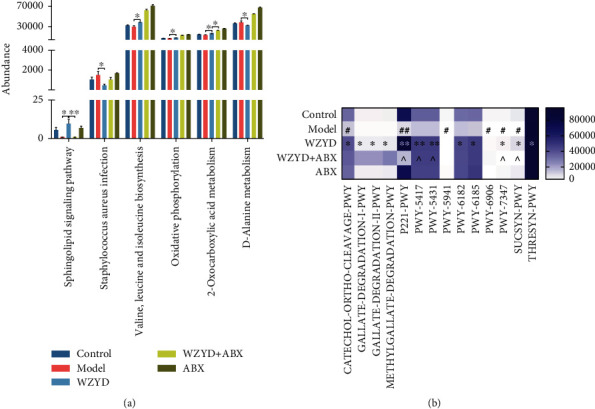
The effects of ABX intervention on WZYD regulating function of microbiota in CM. (a) The analysis of KEGG pathway. ^∗^*P* < 0.05; ^∗∗^*P* < 0.01. (b) Significant changes of metabolic pathway expressed as a heat map. Compared to control group, ^#^*P* < 0.05 and ^##^*P* < 0.01; compared to model group, ^∗^*P* < 0.05, ^∗∗^*P* < 0.01, and ^∗∗∗^*P* < 0.001; compared to WZYD group, ^*P* < 0.05.

Based on the 16S rRNA sequencing data, PICRUSt was used to predict the bacterial genes. And KEGG (Figure 3(a)) and MetaCyc (Figure 3(b)) databases were used to predict functional and metabolic pathway. Compared to the control group, the decreased abundance of KEGG signaling pathway were biofilm formation-Pseudomonas aeruginosa (*P* < 0.05); sphingolipid signaling pathway (*P* < 0.01), arginine and proline metabolism (*P* < 0.05); isoquinoline alkaloid biosynthesis (*P* < 0.01), tropane, piperidine, and pyridine alkaloid biosynthesis (*P* < 0.05); fluorobenzoate degradation (*P* < 0.05); melanogenesis (*P* < 0.05); the increased abundances were RNA transport (*P* < 0.05), central carbon metabolism in cancer (*P* < 0.05), penicillin and cephalosporin biosynthesis (*P* < 0.05), galactose metabolism (*P* < 0.05), and methane metabolism (*P* < 0.05) in the model group. Compared to the model group, the increased abundances of KEGG signaling pathway were sphingolipid signaling pathway (*P* < 0.05), valine, leucine and isoleucine biosynthesis (*P* < 0.05), oxidative phosphorylation (*P* < 0.05), and 2-oxocarboxylic acid metabolism (*P* < 0.05), and the decreased abundances were Staphylococcus aureus infection (*P* < 0.05) and D-alanine metabolism (*P* < 0.05) in the WZYD group. Compared to the WZYD group, the abundance of sphingolipid signaling pathway was significantly decreased after WZYD+ABX treatment (*P* < 0.01).

The analysis of metabolism pathway was shown as a heat map in Figure 3(b). Compared to the control group, the abundances of CATECHOL-ORTHO-CLEAVAGE-PWY, P221-PWY (octane oxidation), PWY-5941 (glycogen degradation), PWY-6909 (thiazole component of thiamine diphosphate biosynthesis III), PWY-7347 (sucrose biosynthesis III), and SUCSYN-PWY (sucrose biosynthesis I) were decreased in the model group. Among them, the abundances of CATECHOL-ORTHO-CLEAVAGE-PWY, P221-PWY, PWY-7347, and SUCSYN-PWY were recovered after WZYD treatment. Meanwhile, the abundances of GALLATE-DEGRADATION-I-PWY, GALLATE-DEGRADATION-II-PWY, METHYLGALLATE-DEGRADATION-PWY, PWY-5417 (catechol degradation III), PWY-5431 (aromatic compounds' degradation via *β*-ketoadipate), PWY-6182 (superpathway of salicylate degradation), PWY-6185 (4-methylcatechol degradation), and THRESYN-PWY (superpathway of L-threonine biosynthesis) were increased. Compared to the WZYD group, the abundances of P221-PWY, PWY-5417, PWY-5431, PWY-7347, and SUCSYN-PWY were decreased in the WZYD+ABX group.

### 3.4. Role of Gut Microbiota in WZYD Downregulating CGRP, Inflammatory Factor, and c-Fos

**Figure 4:**
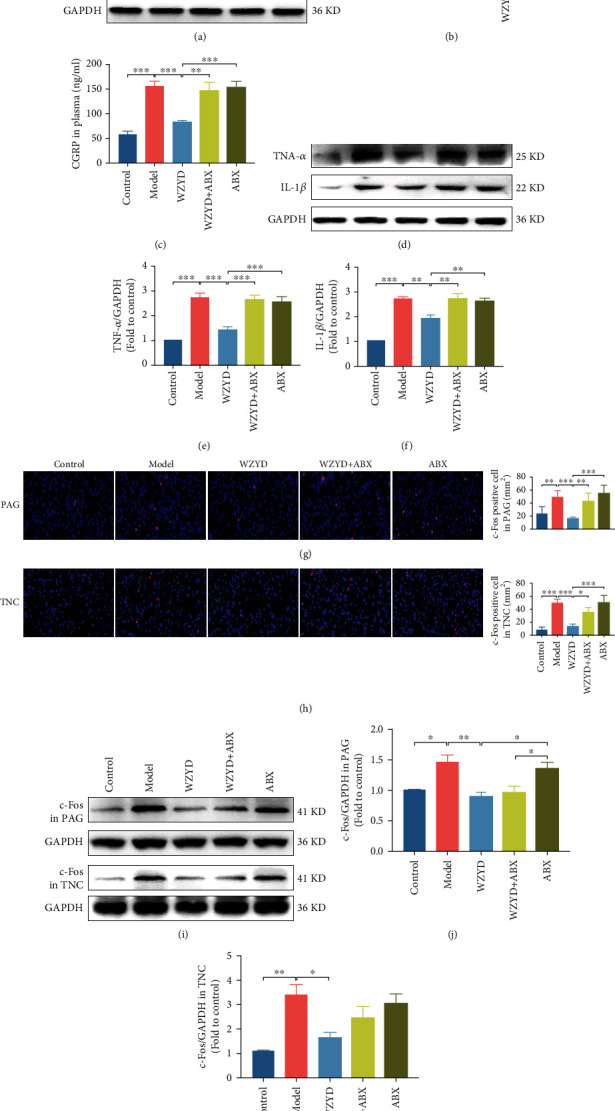
The gut microbiota mediated the effects of WZYD on CGRP, inflammatory factor, and c-Fos. (a, b) The protein expression of CGRP in TNC. (c) The content of CGRP in plasma. (d–f) The protein expressions of TNF-*α* and IL-1*β* in TNC. (g) The c-Fos positive cell in PAG by IF staining. (h) The c-Fos positive cell in TNC by IF staining. (i–k) The protein expression of c-Fos in PAG and TNC. ^∗^*P* < 0.05, ^∗∗^*P* < 0.01, and ^∗∗∗^*P* < 0.001.

The CGRP was examined whether the effect of WZYD would be changed by gut microbiota. The level of CGRP in TNC and plasma was significantly reduced after WZYD treatment compared to model group (*P* < 0.001; Figures 4(a)–4(c)). The clarity of gut microbiota led to a diminished effect of WZYD. Certainly, ABX had no effect for migraine attacks. The protein expressions of TNF-*α* and IL-1*β* were increased in response to repeated IS administration. The effects of WZYD on anti-inflammatory factors could also be affected by gut microbiota. Unexpectedly, ABX which eliminated the intestinal microbiota could not suppress neurogenic inflammation (Figures 4(d)–4(f)). The expression level of c-Fos was evaluated by IF staining and WB. Compared to the control group, the numbers of c-Fos positive cell were increased in TNC (*P* < 0.01; Figure 4(g)) and PAG (*P* < 0.001; Figure 4(h)). The effects of WZYD on c-Fos could be diminished by ABX. The similar results were obtained after WB assay (Figures 4(i)–4(k)).

### 3.5. Role of Gut Microbiota in WZYD Upregulating 5-HT, 5-HIAA, and Related Protein in Brainstem

**Figure 5:**
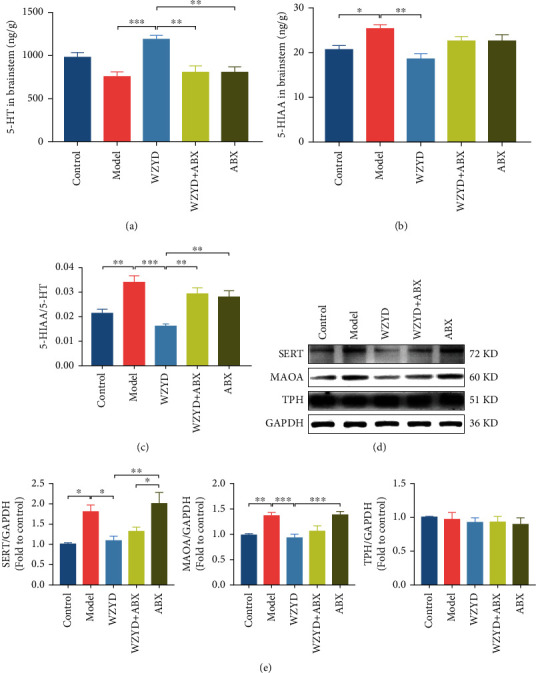
The gut microbiota mediated the effects of WZYD on 5-HT, 5-HIAA, and related protein in brainstem. (a) 5-HT in brainstem. (b) 5-HIAA in brainstem. (c) 5-HIAA/5-HT. (d, e) The protein expression of SERT, MAOA, and TPH. ^∗^*P* < 0.05, ^∗∗^*P* < 0.01, and ^∗∗∗^*P* < 0.001.

5-HT is the key neurotransmitter of descending nociceptive inhibition system in central nervous system. 5-HT was increased (Figure 5(a)) but no change of TPH expression after WZYD administration (Figures 5(d) and 5(e)). 5-HIAA is the metabolite of 5-HT. Compared to the control group, the contents of 5-HIAA (Figure 5(b)), 5-HIAA/5-HT (Figure 5(c)), and the expressions of SERT and MAOA (Figures 5(d) and 5(e)) were increased in the model group, which suggested increasing of 5-HT reuptake and metabolism. The treatment of WZYD decreased the metabolism of 5-HT to 5-HIAA. ABX application could not only increase 5-HT synthesis but also inhibit the therapeutic effect of WZYD.

### 3.6. Role of Gut Microbiota in WZYD Upregulating 5-HT and Neuronal Cell in Hippocampus

**Figure 6:**
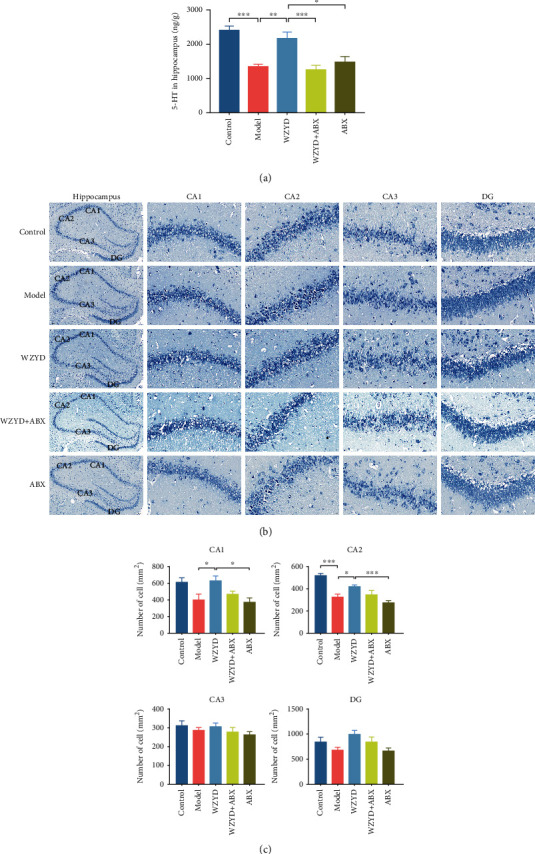
The gut microbiota mediated the effects of WZYD on 5-HT and neuronal cell in hippocampus. (a) 5-HT in hippocampus. (b, c) The neuronal cell in hippocampus of CA1, CA2, CA3, and DG regions. ^∗^*P* < 0.05, ^∗∗^*P* < 0.01, and ^∗∗∗^*P* < 0.001.

The previous behavioral tests showed depression and anxiety in CM model rats associated to 5-HT (Figure 6(a); *P* < 0.001) and neuronal cell decreasing (Figures 6(b) and 6(c); *P* < 0.001 of CA2 region) in hippocampus. The treatment of WZYD increased 5-HT level (*P* < 0.01) and number of neuronal cell (*P* < 0.05 of CA1 and CA2 region). By contrast, ABX administration reversed the increasing of hippocampal 5-HT caused by WZYD treatment. Compared to WZYD group, there were no effects of ABX group on increasing 5-HT and neuronal cell.

### 3.7. Role of Gut Microbiota in WZYD Upregulating 5-HT and Related Protein in Colon Specimens

**Figure 7:**
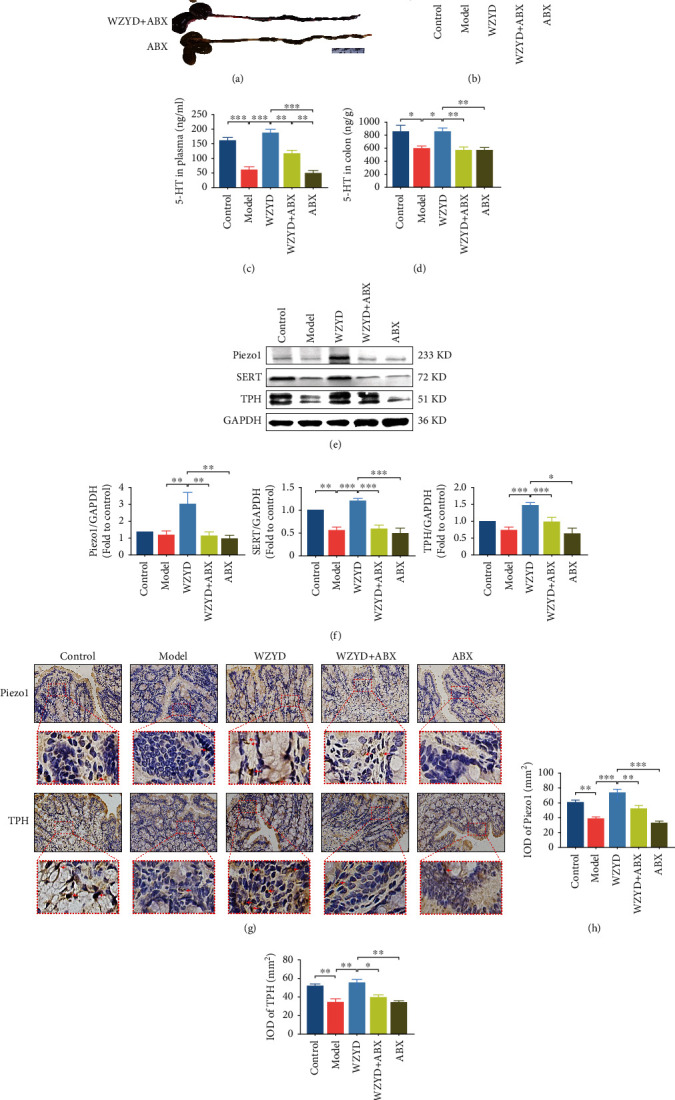
The gut microbiota mediated the effects of WZYD on length, peripheral 5-HT, and related protein in colon. (a) The morphology of colon specimens. (b) The statistic of colon length. (c) 5-HT content in plasma. (d) 5-HT content in colon specimens. (e, f) The protein expression of SERT and TPH by WB. (g–i) The positive expression of Piezo1 and TPH by IHC. Brown-yellow particles marked by the red arrows are the positive expression of Piezo1 and TPH. ^∗^*P* < 0.05, ^∗∗^*P* < 0.01, and ^∗∗∗^*P* < 0.001.

For the measurement of rat colon length, it was found that the CM model and WZYD treatment rats had no significant effect on colon length, while the cecum volume, colon length, and fecal retention increased, and colonic peristalsis was slower after ABX treatment (Figures 7(a) and 7(b)). 5-HT was also determined in plasma and colon specimens (Figures 7(c) and 7(d)). The 5-HT contents were both reduced in model group (*P* < 0.001; *P* < 0.05) and increased in WZYD group (*P* < 0.001; *P* < 0.05). The application of ABX + WZYD counteracted the effect of WZYD in plasma (*P* < 0.01). In order to study the effect of WZYD and ABX on the colon for 5-HT synthesis and release, the expression of Piezo1, TPH, and SERT were detected by WB (Figures 7(e) and 7(f)) or IHC (Figures 7(g)–7(i)). It was found that these protein expressions were significantly increased after WZYD treatment and decreased in ABX+WZYD group.

### 3.8. Role of Gut Microbiota in WZYD Upregulating EC Cells in Colon Specimens

**Figure 8:**
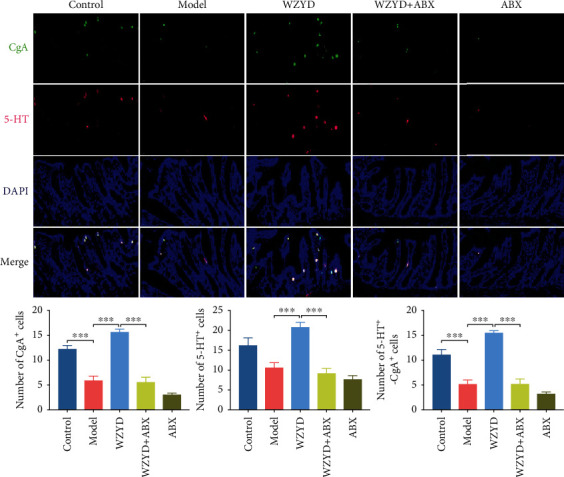
The gut microbiota mediated the effects of WZYD on numbers of CgA and 5-HT positive cells in CM. ^∗∗∗^*P* < 0.001.

The CgA^+^ and 5-HT^+^ cells in colon were shown by IF staining (Figure 8). Compared to control group, significant reductions of CgA^+^, CgA^+^, and CgA-5-HT^+^ cells were shown in model group. After WZYD treatment, the numbers of CgA^+^, 5-HT^+^, and CgA-5-HT^+^ cells were higher than model group. And ABX intervention could weaken WZYD effects.

## 4. Discussion

As a recurrent, highly disabling neurological disorder, the treatment of some drugs may make the attack frequency in CM. The traditional Chinese medicine has clinical experience and efficacy for thousands of years, and a number of studies have clarified their potential for CM. In previous studies, WZYD was found to ameliorate CM by increasing 5-HT and norepinephrine [11], and the gut microbiota also changed during the disease and treatment [7]. Based on the above findings, in order to clarify the roles of gut microbiota in WZYD treatment migraine, the efficacy of WZYD on IS-induced CM model rats associated with gut microbiota depletion was observed in this study.

On the basis of repeat IS stimulation to the dura, the hyperalgesia was significantly increased in the model group. The treatment of WZYD not only decreased the expression of CGRP and c-Fos but also regulated the content of 5-HIAA/5-HT, MAOA, and SERT in the center and increased the contents of 5-HT, TPH, Piezo1, and SERT in the periphery. Part of the results was similar to previous study [11, 14]. Moreover it was found that WZYD elevated central 5-HT through the inhibition of 5-HT metabolism in this study. However, ABX administration combined with WZYD reversed the therapeutic effect of WZYD to some extent. These results indicated that gut microbiota played an important role in WZYD treatment of CM. In conclusion, WZYD relieved hyperalgesia by regulating 5-HT synthesis and metabolism, which might base on gut microbiota.

In this study, the rats in the model group also exhibited anxiety-depression like behavior with reduction of 5-HT and neuronal numbers in the hippocampus, which were improved after WZYD treatment. In another study of CM models, most rats exhibited depression and anxiety-like behaviors [5]. The damaged hippocampal neurons by chronic stress induced the number of neurons decreasing or specimen atrophy leading to depression [15]. Migraine and depression are two highly prevalent diseases in the world, which cause severely disability and loss of quality of life. Previous studies had found a higher prevalence of major depressive disorder (MDD) in migraine patients [16]. Antidepressants are commonly used as prophylactic for migraine [17], while onabotulinumtoxin A for migraine is equally effective in depression [16]. However, the exact biological mechanism of the comorbidity between migraine and depression is unclear. In the past decades, 5-HT has been a crucial neurotransmitter in the pathophysiology of both diseases.

It is widely known that probiotics could degrade fibers that were difficult to be digested by the host and produce secondary bile acids and short-chain fatty acids (SCFAs), participating in the Trp metabolism and 5-HT generation. Some researchers found that the frequency and intensity of migraine attacks can be reduced to varying degrees by probiotics administration and that partial colonized bacteria in the gut microbiota could regulate the 5-HT biosynthesis in the host [18]. In addition, the relationship between gut microbiota and anxiety-depression has also been widely concerned. Compared to the healthy people, the richness and diversity of gut microbiota in patients with mental disorders and the bacteria producing SCFAs were significantly reduced, and the numbers of *Escherichia-Shigella*, *Fusobacterium*, and *Ruminococcus gnavus* were increased [19]. The germ-free mice were more prone to reduced 5-HT synthesis and increased depression-like behavior [20], and the intestinal colonization of symbiotic flora could increase monoamine neurotransmitters in the brain and normalize anxiety-like behavior [21]. The results of microbiota analysis showed that the application of ABX significantly reduced the probiotics in feces, increased the bacteria resistant to ABX as well as fecal retention and soft defecation, affected neurogenesis in the hippocampus, and led anxiety-depression like behaviors [22]. Compared to the WZYD group, pain threshold decreased, the expression of CGRP and inflammatory factors increased, the levels of 5-HT reduced with metabolism increased, and production decreased in the WZYD+ABX group. It indicated that the effect of WZYD was weakened and the important role of gut microbiota in the treatment of migraine with WZYD. The results of a clinical study conducted by Talbott et al. showed that there was a close relationship between microbial community balance and psychological parameters. It was emphasized that targeted supplementation of probiotics was beneficial to optimize the balance of gut-brain axis to enhance mental health [23].

The gut microbiota plays an important role in host health, which can prevent pathogen colonization, regulate intestinal immunity, provide essential nutrients and active metabolites, and participate in energy homeostasis [24]. In this study, the fecal microbiota sequencing analysis found that there was no difference in the abundance of the first three phyla in the model group and WZYD group. ABX administration made the proteobacteria became the main dominant bacteria, which included many pathogens, such as E. coli, Salmonella, Vibrio cholerae, and Helicobacter pylori, and reflected microdysbiosis or unstable gut microbial community structure. A significant increase in both Acinetobacter and Butyricicoccus was analyzed at genus level in the WZYD group, while the latter one was a butyrate producer and had a role in promoting SCFA generation [25]. Enterobacter as an opportunistic pathogen increased significantly after treatment with WZYD+ABX and associated with decreased SCFA generation [26].The abundance of *Holdemania* was positively related with the generation of SCFA, EC cells, and 5-HT [27]. Moreover, the abundances of genera promoted SCFA synthesis were also significantly reduced, for example, Coprococcus, Prevotellaceae_Prevotella [28], and Eubacterium [29] in the WZYD+ABX group.

The KEGG signaling pathway analysis found that the treatment of WZYD had the restoration of the sphingolipid signaling pathway in the CM model rats, which was counteracted by ABX. Subtle changes in sphingolipid balance might be associated to neurological disorders and involved in pain-related neuronal function and signaling pathways. By detecting the concentration of sphingolipids in serum and cerebrospinal fluid of patients with migraine attacks, it was used to judge migraine attacks of the decreased total ceramide and dihydroceramide [30]. The metabolic pathways regulated by WZYD treatment, such as PWY-7347 and SUCSYN-PWY, were reversed in the WZYD+ABX group, which were related to sucrose biosynthesis, which were reported of analgesia in newborn infants undergoing painful procedure [31].

However, the study had only analyzed microbial signature of dysbiosis in gut microbiota. It was not determined which bacteria induced CM attack, nor which bacteria were affected by WZYD treatment. It also might be the interaction among multiple bacteria, which still needs to continue the relevant studies for analysis and to explain the relationship between gut microbiota and migraine of mechanism and treatment.

## 5. Conclusion

In conclusion, our study demonstrates that the WZYD treatment improved CM hyperalgesia dependent on the presence and stability of the intestinal microbiota. Furthermore, WZYD may ameliorate anxiety-depression like behavior associated with migraine. Our study provides new basic evidence for the mechanism of WZYD in migraine treatment and provides important guidance for future clinical research and effective treatment.

## Data Availability

The raw figures and statistic data used to support the findings of this study are available from the corresponding author upon request.

## Supplementary Materials

Supplementary 1 Supplementary 1: the sources and amounts of raw materials and the contents of active ingredients.

Supplementary 2 Supplementary 2: primary antibodies.
