# Supplementary material for: The Microbiota-Dependent Treatment of Wuzhuyu Decoction for Chronic Migraine Model Rat Associated with Anxiety-Depression Like Behavior
Source: Oxid Med Cell Longev. 2023 Jan 6;2023:2302653. doi: 10.1155/2023/2302653 (PMC9840058; doi:10.1155/2023/2302653)
Supplement: Supplementary 1 — the sources and amounts of raw materials and the contents of active ingredients. [file 2302653.f1.docx]

The herbal samples were retained in the Chinese Medicine Pharmacy Laboratory, School of Traditional Chinese Medicine, Capital Medical University. The sample number is shown in the Table 1.

Table 1 The sources, identification numbers and amounts of 4 herbal ingredients

| Medicinal materials | Euodiae Fructus (Wuzhuyu) | Ginseng Radix et Rhizoma (Renshen) | Jujubae Fructus (Dazao) | Zingiberis Rhizoma Recens (Shengjiang) |
| --- | --- | --- | --- | --- |
| Identification numbers (No.) | 201705001-WZY | 201705001-RS | 201705001-DZ | 201705001-SJ |
| Sources | Jiangxi, China | Jilin, China | Xinjiang, China | Beijing, China |
| Amounts | 54g | 54g | 64g | 108g |

According to the previous methods ^[^^[[1]](#endnote-1)]^, the percentage content of components was analyzed by HPLC in Euodiae Fructus, Ginseng Radix et Rhizoma and Zingiberis Rhizoma Recens. According to the determination of Pharmacopoeia, Li content should not be less than 0.20%, the sum of Ev and Ru content should not be less than 0.15%; the sum content of Rg1 and Re should not be less than 0.30%, Rb1 content should not be less than 0.20%, and 6-Gi content should not be less than 0.05%. All the ingredient used in this research were complied with the provisions of the Pharmacopoeia.

Table 2 The percentage contents of 7 components in Euodiae Fructus, Ginseng Radix et Rhizoma and Zingiberis Rhizoma Recens.

| Medicinal material | Li | Ev | Ru | Medicinal materials | Rg1 | Re | Rb1 | Medicinal materials | 6-Gi |
| --- | --- | --- | --- | --- | --- | --- | --- | --- | --- |
| Euodiae Fructus | 0.54 | 2.00 | 0.81 | Ginseng Radix et Rhizoma | 0.21 | 0.27 | 0.3 | Zingiberis Rhizoma Recens | 0.12 |

1. [] Xu Yongsong. Recognition and optimization of anti-migrainic ingredients from Wuzhuyu decoction based on intestinal absorption spectrum-effect correlation[D]. Capital Medical University, 2019. [↑](#endnote-ref-1)
