# Supplementary material for: The Microbiota-Dependent Treatment of Wuzhuyu Decoction for Chronic Migraine Model Rat Associated with Anxiety-Depression Like Behavior
Source: Oxid Med Cell Longev. 2023 Jan 6;2023:2302653. doi: 10.1155/2023/2302653 (PMC9840058; doi:10.1155/2023/2302653)
Supplement: Supplementary 2 — primary antibodies. [file 2302653.f2.docx]

| Antibodies | Molecular weight | No./Brand | Host | Dilution ratio |
| --- | --- | --- | --- | --- |
| c-fos | 50-65 | ab190289/Abcam | Rabbit | 1:1000 |
| CGRP | 14 | ab139264/ Abcam | Rabbit | 1:1000 |
| TNF-α | 25/28 | ab66579/ Abcam | Rabbit | 1:1000 |
| IL-1β | 22 | sc-12742/Santa Cruz | Rabbit | 1:200 |
| TPH | 51 | ab52954/Abcam | Rabbit | 1:1000 |
| SERT | 72 | ab272912/Abcam | Rabbit | 1:1000 |
| Piezo1 | 286 | 15939-1-AP/Proteintech | Rabbit | 1:500 |
| MAOA | 60 | Ab126751/Abcam | Rabbit | 1:1000 |
| GAPDH | 36 | 60004-1-lg/Proteintech | Mouse | 1:10000 |
|  |  |  |  |  |
